# Effect of protein modification in synbiotic infant formula on probiotic metabolic activity and bacterial composition in an infant gut-model

**DOI:** 10.20517/mrr.2024.13

**Published:** 2024-06-28

**Authors:** Eline Suzanne Klaassens, Mirna Lilian Baak, Nienke Jacobine Mekkes, Radhika Bongoni, Monika Schaubeck

**Affiliations:** ^1^BaseClear B.V., Leiden 2333 BE, the Netherlands.; ^2^Research & Development, HiPP GmbH & Co. Vertrieb KG, Pfaffenhofen 85276, Germany.

**Keywords:** Infant microbiome, metagenomics, probiotics, hydrolyzed, *Limosilactobacillus*, * Bifidobacterium*

## Abstract

**Aim:** Microbial colonization of the neonatal gut is pivotal in priming the infant’s immune system. Human milk (HM) is the best nutrition for infants and supports the development of the microbiota due to prebiotic compounds and probiotic microorganisms. When exclusive breastfeeding is not possible, infant formula (IF) with probiotics is a strategy to support the infant’s microbiome development. However, knowledge about the effects of the infant gut microbiota and different compounds in IF on individual probiotic strains is limited, as strain-level detection in a complex ecosystem is challenging. The aim of the present study was to show the effects of IF with different protein forms on the metabolic activity of two probiotic strains isolated from HM in a complex ecosystem.

**Methods:** By using an *ex-vivo* infant gut model containing infant donor-microbiota, the effects of IF with either intact or extensively hydrolyzed protein on the metabolic activity of the donor microbiota, as well as two probiotic strains [*Limosilactobacillus fermentum* (*L. fermentum*) CECT 5716 (Lf) and *Bifidobacterium breve* (*B. breve*) DSM 32583 (Bb)], were analyzed. A new bioinformatic pipeline combined with a specific infant microbiome database was used to explore shotgun metagenome datasets (1200 Megabases) for taxonomic identification and strain-level tracking.

**Results:** Both protein forms (i.e., intact or extensively hydrolyzed protein) in IF supported infant gut microbial metabolic activity equally, as evidenced by similar levels of short-chain fatty acids (SCFAs). Interestingly, gut microbial metabolic activity was found to be differently activated in a strain-dependent manner. Taxonomic profiling of the microbiome at the strain level enabled monitoring of the prevalence and abundance of both probiotic strains, even in a complex ecosystem.

**Conclusion:** Food matrix and host microbiota interactions should be considered when evaluating strain-specific probiotic effects in the future.

## INTRODUCTION

In the first days of life, neonates are rapidly colonized by different microorganisms at different body sites. The composition of newly formed microbial ecosystems is mostly influenced by the mode of birth, administration of antibiotics, and nutrition^[1,2]^. By disturbing the microbiota in this early time, the initiation of a physiologic interaction of colonizing microorganisms with the infant’s immune system might be off-balanced^[3]^, and consequently, the risk of infections and allergies might increase^[4]^.

Human milk (HM) is the best nutrition for infants as it ensures optimal growth and development, while also supporting the infant’s microbiota maturation with its probiotic and prebiotic compounds^[5]^. Breastfed infants have a lower risk for infections, metabolic diseases, and other pathologies, presumably also attributed to an age-tailored microbiota^[6]^. The composition of HM macronutrients, micronutrients, and microorganisms changes over the lactation period^[7-9]^. HM is also a source of microorganisms that can subsequently be found in the infant’s intestinal microbiota. However, the extent to which these HM-derived strains contribute to the infant’s microbiota over time is still under debate^[10]^. This might also be due to missing tools to follow these microorganisms at the strain level.

When exclusive breastfeeding is not possible, infant formula (IF) is a dietary substitute for infants. The IF aims to support the nutritional needs, as well as the development of the infant and its developing intestinal microbial ecosystem. Adding probiotic and prebiotic compounds to IF is a possible way to enhance the healthy development of the infant gut microbiota. The safety of the probiotic strain has to be proven and the beneficial effects should be traced back to the specific strain(s)^[11]^. When exclusive breastfeeding is not possible, and the infant is at higher risk for allergy development (e.g., by parental predisposition), some guidelines recommend feeding an IF with hydrolyzed protein as a possible substitute^[12]^. Recently, compounds in IF other than probiotics and prebiotics, such as hydrolyzed protein, were studied for their effect on the infant’s microbiota or immune system^[13-17]^. Additionally, the effect of different IF matrices on the respective formulas’ allergenic activity was tested in an *in vitro* basophil activation experiment. This experiment revealed that probiotic-induced reduction in basophilic degranulation was strongest when the probiotic strain was combined with extensively hydrolyzed protein IF. Thus, these results demonstrate that the protein matrix can have a significant effect on the viability and metabolic activity of the probiotic strains^[17]^.

Being aware of the complex dynamics between the gut microbiota, probiotics and macronutrients such as proteins, we investigated the effects of IF based on extensively hydrolyzed protein and IF with intact (i.e., non-hydrolyzed) proteins, as well as their interactions and colonization benefits to the added probiotics *Bifidobacterium breve* (*B. breve*) DSM 32583 (Bb) and *Limosilactobacillus fermentum* (*L. fermentum*) CECT 5716 (Lf). The two probiotics isolated from HM were studied *in vitro* for their growth and metabolic activity in either extensively hydrolyzed formula (eHF) or intact protein formula (iPF). In order to investigate the effects of two probiotic strains and two different forms of IF on complex gut ecosystems, we used an *ex vivo* digestive system with infant donor microbiota samples. This enabled us to test the very same microbial ecosystem with different interventions and to abstain from animal trials. This approach facilitates the demonstration of the causal effects of nutrition on microbiome development, circumventing some confounding factors and species-specific differences frequently observed in human and murine studies, respectively. Given the lack of precision in current methods for monitoring the abundance of a probiotic strain, a specific infant-microbiota-based pipeline was developed to track the quantity of the probiotic during the different interventions. This bioinformatics pipeline was developed for the analysis of metagenome data with an infant microbiome-specific database. It has been validated for strain-level identification of infant gut metagenomes as well as its capacity for strain tracking and colonization.

## METHODS

### Composition of the IF matrices

IF manufactured from intact cow’s milk protein with a protein content of 1.9 g/100 mL without prebiotics (iPF) served as control formula. iPF with 0.3 g/100 mL galactooligosaccharides (GOS) was used to test the effect of GOS. Additionally, IF with extensively hydrolyzed protein from whey (eHF) with a protein content of 1.9 g/100 mL enriched with 0.3 g/100 mL GOS was used to test the additional effect of a hydrolyzed protein matrix (eHF + GOS). Quantities of protein and other nutrients in both IFs were comparable. All products were in accordance with EC Directive 2006/141/EC.

### *In vitro* analyses in IF

The *in vitro* experiments were carried out by ProbiSearch SLU, Madrid, Spain. Overnight cultures of probiotics in MRScys [de man, Rogosa, Sharpe, Oxoid, (Basingstoke, England) + 0.25% L-cysteine hydrochloride hydrate (Sigma-Aldrich, Steinheim, Germany)] were used to prepare the initial inoculum. Each culture was adjusted with an initial bacterial inoculum (10^9^ CFU/mL) in the respective formula.

Briefly, for a final volume of 330 mL, MilliQ water was autoclaved with a magnetic piece inside. 40.8 g of formula powder were suspended in the autoclaved water just before the beginning of the experiment and then the formula was inoculated with the optical density-adjusted strains. 3.3 mL of each strain inoculum were used. Inoculated formula bottles were mixed for 5 min in a magnetic stirrer. After mixing the inoculum with the formula, 30 tubes with 10 mL each, were filled.

Experiments were performed in triplicates (for Lf, Bb and a combination of Lf + Bb, respectively). Probiotics in IF were plated in serial dilutions to do bacterial counts (incubated at 37 °C). For the quantification of bacteria, serial dilutions were done in peptone water (Oxoid) and plated on MRScys (Oxoid) and TOS (Merk) agar plates. Agar plates were incubated for 48 h. TOS plates, to detect Bb strain, were incubated in anaerobic conditions and MRScys plates, to detect Lf strain, in aerobic conditions. Anaerobic incubations were done in a Whitley DG250 Anaerobic Workstation (Don Whitley Scientific; 5% CO_2_, 10% H_2_, 85% N_2_).

For analysis of gas formation, syringes were filled with 20 mL inoculated IF and closed with a Leuer cap. In addition, one aliquot per syringe was plated to perform bacterial counts at time 0. Then, all the syringes were incubated at 37 °C in anaerobic conditions. The anaerobic incubations were conducted in a Whitley DG250 Anaerobic Workstation (Don Whitley Scientific), with a gas composition of 5% CO_2_, 10% H_2_, and 85% N_2_. At the incubation time points of 0, 8, 24, and 48 h, the total volume of each syringe was recorded.

#### Short-chain fatty acid determination

For the *in vitro* experiments, short-chain fatty acid (SCFA) quantification has been performed by Probisearch SLU (Spain). Quantities of SCFAs were analyzed by gas chromatography (GS-MS), using the column Supelcowax 10 (Supelco) and the following analytical standards: Acetic acid (Sigma), Butanoic acid (Sigma), Propionic acid (Sigma), and 4Methylpentanoic acid as internal standard (IS) (Sigma). Samples were analyzed immediately to avoid any acid loss. All samples were adjusted to a pH range between 3-3.5 in order to coagulate the sample, resulting in separation into two phases: solid phase (discarded) and aqueous liquid phase, which is the target phase. The target phase was enriched with the IS at a final concentration of 100 mg/L. Linearity was evaluated by preparing three calibration curves of acid acetic, propionic acid, and butyric acid, respectively, in an aqueous matrix, with 100 mg/L of isobutyl acetic acid as IS. Recovery coefficients of studied SCFAs were measured in order to evaluate the complete method, by spiking in each matrix at medium-level concentrations (Medium level concentration: Acetic acid: 150 mg/L; propionic and butyric acids 10 mg/L). A GCMS Agilent Technology was used with a short wax column to separate the SCFAs. Temperatures were increased from 40 to 240 °C in 36 min. In the GCMS analysis, the identification of detected compounds is performed in manual mode, by comparing their mass spectra with those available in the database of the library (library Wiley Nist 9 + 8) W9N8.

For the *ex vivo* experiments, SCFA quantification has been carried out by ProDigest (Belgium) in accordance with the methodology described by De Weirdt *et al.*^[18]^. The gas phase composition was analyzed with a Compact GC4.0 (Global Analyser Solutions, Breda, the Netherlands), equipped with a Molsieve 5A pre-column and Porabond Q column (CH_4_, O_2_, H_2_ and N_2_) and a Rt-Q-bond pre-column and column (CO_2_, N_2_O and H_2_S). Concentrations of gases were determined by means of a thermal conductivity detector.

### SHIME^®^ gut model with infant fecal microbiome

The *ex-vivo* experiments were carried out by ProDigest, Ghent, Belgium. Formula matrix-dependent effects were tested in an *in vitro* simulator model of the human digestion system*.* Therefore, the Simulator of the Human Intestinal Microbial Ecosystem (SHIME)^®^ model was used as a multi-compartment dynamic simulator of the human gut^[19]^. The SHIME^®^ model mimics different segments of the gastrointestinal tract and the colon, so that detailed information on the fermentation profile, including the localization of the effects along the intestinal tract, can be assessed. Setup conditions and nutritional media were used according to^[20,21]^. The nutritional medium was setup according to Marsaux *et al.* (final concentration of K_2_HPO_4_ 4.7 g/L; KH_2_PO_4_ 14.7 g/L; NaHCO_3_ 1.8 g/L; yeast extract 1.8 g/L; peptone 1.8 g/L; mucin 0.9 g/L; cysteine 0.5 g/L; polyoxyethylene^[20]^ sorbitan monooleate 1.8 mL/L in the reactors)^[22]^. To show microbiota-inherent metabolic activity, a setup without the addition of probiotics (Blank) was also tested.

The infant donor microbiota from 9 exclusively breastfed infants - 4 of which were born via cesarean section (CS) and 5 of which were born by vaginal delivery (VD) - were used. Fecal material was collected with parental consent and ethical approval of the University Hospital Ghent (reference number B670201836585). To give a general overview of the kinetic of metabolic activity, samples were taken at baseline, as well as after 6, 24, and 48 h of colonic fermentation with the donor microbiota.

IF with intact or hydrolyzed protein were subjected to a full passage through the oral, gastric, and small intestinal phases, the latter involving absorption as described previously^[23]^. Fecal suspensions were prepared in phosphate buffer, to which reducing agents were added (K_2_HPO_4_ 8.8 g/L; KH_2_PO_4_ 6.8 g/L; sodium thioglycolate 0.1 g/L; sodium dithionite 0.015 g/L). The obtained suspensions were mixed with a cryoprotectant [modified version of^[24]^ in a 1:1 (v:v) ratio, so that 7.5% fecal suspensions were obtained]. The fecal suspensions were flash-frozen and then preserved at -80 °C (cryostock). Just before an experiment, fecal samples were defrosted and immediately added to the reactors. At the start of the short-term colonic incubation, the test ingredients (pre-digested infant formulae and probiotic strains) were added to sugar-depleted buffered nutritional medium containing basal nutrients present in the colon. Probiotic strains were added in a concentration of 1.5 × 10^7^ CFU/mL each. Finally, the cryopreserved fecal suspensions of each of the donors were added [10% (v:v)]. Control experiments had no probiotic addition (Blank). Reactors were incubated for 48 h at 37 °C under continuous shaking (90 rpm) and anaerobic conditions. The incubations were performed in fully independent reactors with sufficiently high volume (70 mL) in order to not only ensure robust microbial fermentation, but also enable the collection of multiple samples over time. Samples were collected for metagenome sequencing and the metagenome data were used to investigate the taxonomic profiles at the species and strain levels. DNA extraction was carried out in accordance with the methodology outlined in Duysburgh *et al.*^[25]^, which was based on the approach proposed by Boon *et al.*^[26]^. DNA QC quantitation was performed with Quant-iT^TM^ dsDNA Broad-Range Assay Kit (Invitrogen) and agarose gel electrophoresis for DNA integrity.

### Metagenome sequencing of the microbiome

DNA samples were subject to Illumina Nextera XT library preparation. The sequencing libraries obtained, with an insert size of at least 300 bp, were sequenced on a NovaSeq 6000 instrument with paired-end 150 nt sequencing protocol on 1 S1 flowcell. Demultiplexed reads were trimmed and subsampled to 1,200 MB. FASTQ read sequence files were generated using bcl2fastq2 version 2.18, which includes Illumina Chastity quality filtering with default settings. Subsequently, reads containing PhiX control signal were removed using Bowtie 2.2.6. In addition, reads containing (partial) adapters were clipped (up to a minimum read length of 50 bp) with ea-utils 1.0.4. The second quality assessment was based on the remaining reads using the FASTQC quality control tool version 0.11.8. The resulting FASTQ files were used as input for taxonomic profiling as described below, using an internal infant-specific reference database V1 as a secondary input. The whole genome sequences (WGS) of both probiotics (Bb and Lf) were added to this database for strain tracking purposes.

### Specific database for taxonomic profiling of metagenome data to strain level in infant-specific intestinal ecosystems

To increase the resolution of taxonomic classification, a specific metagenome database was built with genome assemblies targeted toward the infant gut microbiome. This database was used in a Kraken-Bracken-based assembly-free taxonomic profiling workflow^[27]^. Compiling the database, a literature search was performed to create a list of bacterial genera that are commonly found in the infant gut (09-Dec-2021). We created this list at the family level to ensure the inclusion of all closely related genera. All publicly available genomes belonging to these families were collected by search in Refseq^[28]^, GenBank^[29]^, ENA^[30]^, and DDBJ^[31]^ databases. Only complete and scaffold level assemblies were used, since contig level assemblies are usually too small and low-quality to add to the database. The following genomes were added to the Refseq database: 18,224 genomes of the *Pasteurellales*, *Bacteroidales*, *Clostridiales*, *Enterobacterales*, *Selenomonadales*, and *Verrucomicrobiales* orders, 951 genomes from a recently published human (adult) gut microbiome catalog^[32]^, 2,132 genomes belonging to the archaeal phyla *Crenarchaeota*, *Euryarchaeota*, and *Thaumarchaeota*^[33,34]^, 5,252 Eukaryotic genomes, namely the *Ascomycota*, *Basidiomycota*, *Glomeromycotina*, *and Mucoromycota*^[35]^, and viral genomes from the Human Reference Genome (GRCh38.p13) to filter out reads belonging to the human genome, and a list of common vectors (UniCoreVec). After combining all these genomes, any potential duplicates remaining were removed. Genomes of low quality or too short scaffolding length were removed as well. This resulted in a database with a total of 73,085 complete and scaffold-level genomes relevant to the infant gut microbiome. The genome assemblies of probiotic strains used in this study were added to this database, enabling us to trace them in our experimental samples.

We built the Kraken2 V2.1.1^[27]^ database as described in the Kraken reference manual provided online (https://ccb.jhu.edu/software/kraken/MANUAL.html). Inspecting the database after building showed that 99.98% of the minimizers were mapped to the lowest common ancestor (LCA) (see graphical abstract for a graphical overview of the database).

For the bioinformatics analysis pipeline, we used Kraken 2, together with Bracken. We did not use the standard settings for several steps in these tools, but optimized them for our specific question here. The adaptions were set as follows: for Kraken: Confidence: 0.05; For Bracken: minimum reads (-t) 100; read length (-r) 150; level (-l) D, P, C, O, F ,G ,S, S1.

The number of taxa is smaller than the number of added genomes because many taxa have multiple genomes representing them. A Bracken V2.5^[36]^ database was constructed based on the finished Kraken2 database with a read length of 150 bp.

### Taxonomic profiling pipeline

Nextflow^[37]^ was used to write the infant taxonomic profiling pipeline. A schematic overview of the entire workflow can be found in the graphical abstract. The computational part of the workflow combines the following parts: Read in the paired-end reads in FASTQ format; Paired-end read classification down to the LCA with Kraken2 using a specific infant gut microbiome database; Abundance profiling on different taxonomic levels (from Domain to Strain) using Bracken. Bracken is designed to redistribute reads classified by Kraken2. By default, Bracken only redistributes down to the species level. However, it is possible to redistribute all the species-level reads down to the subspecies and strain levels. Redistribution at the strain level is performed based on the assigned reads and available kmers per strain by Kraken2 using Bracken (bayesian estimation of abundance with Kraken)^[36]^.

### Bioinformatics analysis of metagenomic sequencing data

After sequencing and demultiplexing the samples, the FASTQ files were analyzed in the following way: The bioinformatics pipeline described above was run using a Kraken2 confidence of 0.1 and a Bracken threshold of 100 reads.

The relative abundance was adjusted for the number of reads classified by Kraken2 and accepted by Bracken. The relative read abundances were then used to analyze the composition of every sample at the genus, species and strain levels. Figures were created within R3.6.0 [R Core Team (2021) using vegan (version 2.5-5)]^[38]^ package and ggplot2 (v3.1.1)^[39]^. Permanova statistics (adonis, vegan package) was performed to investigate whether the centroids and dispersion of the two groups are equivalent.

### Statistical analysis

To assess whether differences between treatment effects in terms of the investigated endpoints were statistically relevant, paired two-sided *t*-tests within a given donor group [(1) vaginal, (2) C-section born, and (3) all donors] were performed. To control the proportion of false discoveries when conducting a high number of comparisons, the Benjamini-Hochberg false discovery rate (FDR) was applied. Differences between treatment effects were considered significant when the obtained *P*-value (obtained through the paired two-sided *t*-test) was smaller than a reference value (ref). This reference value was obtained by ranking of the obtained *P*-values in ascending order within the donor group. The rank of a given *P*-value was termed (i) and varied between 1 and the total amount of *P*-values (m = 9, as mentioned below). The reference value was calculated by multiplying the FDR with the rank of the *P*-value, divided by the total amount of comparisons made (ref = FDR*i/m). To compare the differences between treatment effects in terms of changes in pH levels, gas pressures, and microbial metabolite productions (SCFA, lactate and ammonium), an FDR of 0.1 was used.

Graphs and statistical analyses were generated in Prism version 9.2 (GraphPad, San Diego, CA, US). Graphical abstract was created with BioRender.com.

## RESULTS

### Matrix-dependent effects on probiotic growth and activity *in vitro*

When probiotic strains are combined with IF, the surrounding formula matrix (i.e., the sum of prevailing prebiotics, as well as carbohydrates, fats, and proteins) might have an effect on probiotic growth and activity.

**Figure 1:**
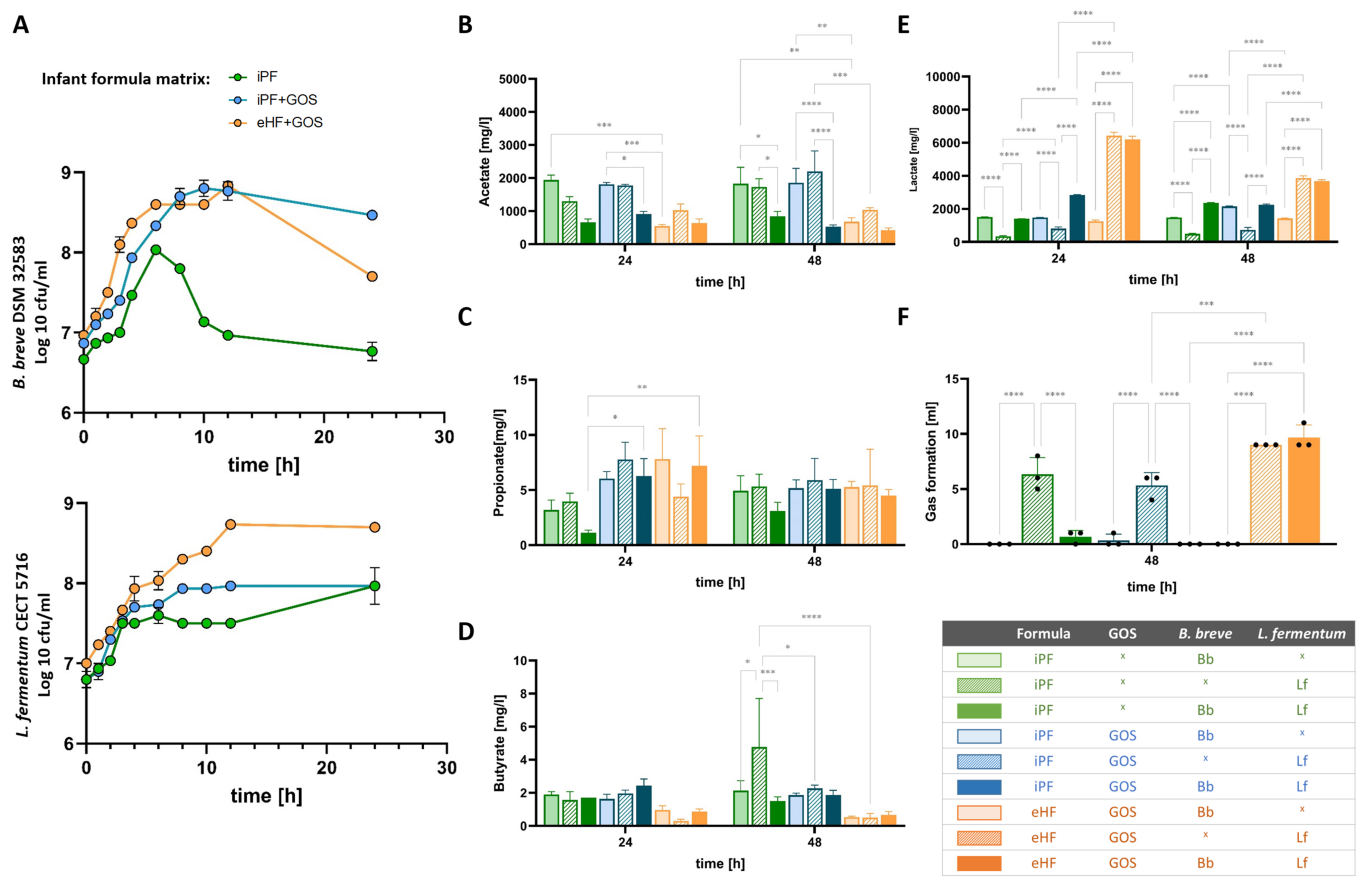
Growth and metabolic activity of probiotic strains in single and co-culture conditions in the presence of prebiotics or intact/hydrolyzed protein. (A) Growth of Bb (top) and Lf (bottom) in co-culture (1:1 ratio), in the presence of iPF without prebiotics (green), with GOS (blue), or eHF with GOS (orange); (B) Formation of Acetate; (C) Propionate; (D) Butyrate; or (E) Lactate or (F) volume of gas formation in ml after 24 and/or 48 h after growth of Bb as single culture (open bars), Lf as single culture (dashed lines bar) or both strains as co-culture (filled bars) in the different formulae. Error shown is the standard deviation. *n* = 3 per time point. Selection of significant differences are marked with a ^*^P < 0.05, ^**^P < 0.01, ^***^P < 0.001, ^****^P < 0.0001. iPF: Intact protein formula; GOS: galactooligosaccharide, eHF: extensively hydrolyzed formula; Bb: *B. breve* DSM 32583; Lf: *L. fermentum* CECT 5716.

The effect of different formula matrices, or the presence of GOS, on the growth rates of Lf and Bb (combined in a 1:1 co-culture) was tested *in vitro*. The addition of GOS increased the growth rates of both strains (Figure 1A; iPF *vs.* iPF + GOS). The eHF + GOS matrix showed strain-specific effects as indicated by differing CFU/mL kinetics between Lf and Bb in the respective formula matrices (Figure 1A, iPF + GOS *vs.* eHF + GOS). In the formula containing extensively hydrolyzed protein and GOS (eHF + GOS), Lf showed significantly higher growth rates compared to the iPF and GOS (iPF + GOS; after 12 h: 8.7 *vs*. 8.0 log10 CFU/mL respectively; *P* < 0.001). For Bb, no growth-supportive effect of the eHF + GOS, compared to iPF + GOS, was seen (8.8 *vs*. 8.8 log10 CFU/mL, respectively; n.s.).

Probiotic activity was visible through the formation of different acids such as acetate, propionate, butyrate, and lactate along the fermentation process. To evaluate the respective amount of SCFA which is produced by either Lf, Bb, or the combination of the strains, the probiotics were either grown as single strains or simultaneously (combined in a 1:1 co-culture). Bacterial growth was comparable between single and co-culture setups (data not shown). In single culture, Bb produced high acetate levels, especially in a matrix with intact protein (Figure 1B; open bars). Acetate production was independent of GOS availability, as shown by the non-significant differences between iPF and iPF + GOS setups. Co-culture of Bb and Lf showed the lowest acetate levels in all tested formula setups and time points (filled bars).

In contrast, propionate production was significantly higher in the presence of GOS, when Bb and Lf were added in a 1:1 ratio (filled bars), independent of the formula matrix [Figure 1C]. Butyrate, however, was highest in formula without prebiotic or eHF, especially for Lf [Figure 1D]. Lactate formation was highest in the eHF + GOS matrix [Figure 1E].

The production of SCFA and microbial fermentation can be associated with gas formation *in vitro*, as shown for Lf in single culture [Figure 1F]. Interestingly, the combined growth with Bb significantly abrogated the amount of gas detected after 48 h, despite growth and associated gas production of Lf in a matrix with iPF. This effect was specific for the iPF only, as gas production in the eHF is not diminished, probably due to a massive decrease in the growth of Bb in eHF + GOS at this time point [Figure 1A].

It could be shown that *in vitro*, both strains were growing well in single and co-cultured conditions. The presence of prebiotics and the protein form influenced the growth and metabolic activity of the probiotic strains *in vitro*. Culturing the strains in single- and co-cultured conditions indicated the presence of cross-feeding mechanisms, as seen in different levels of SCFA and gas volume. Therefore, probiotic activity and abundance of Lf and Bb in the presence of a complex microbiota were assessed in the SHIME^®^ model.

### Effect of infant formula matrix on the metabolic activity of donor infant intestinal microbial ecosystems *ex vivo*

**Figure 2:**
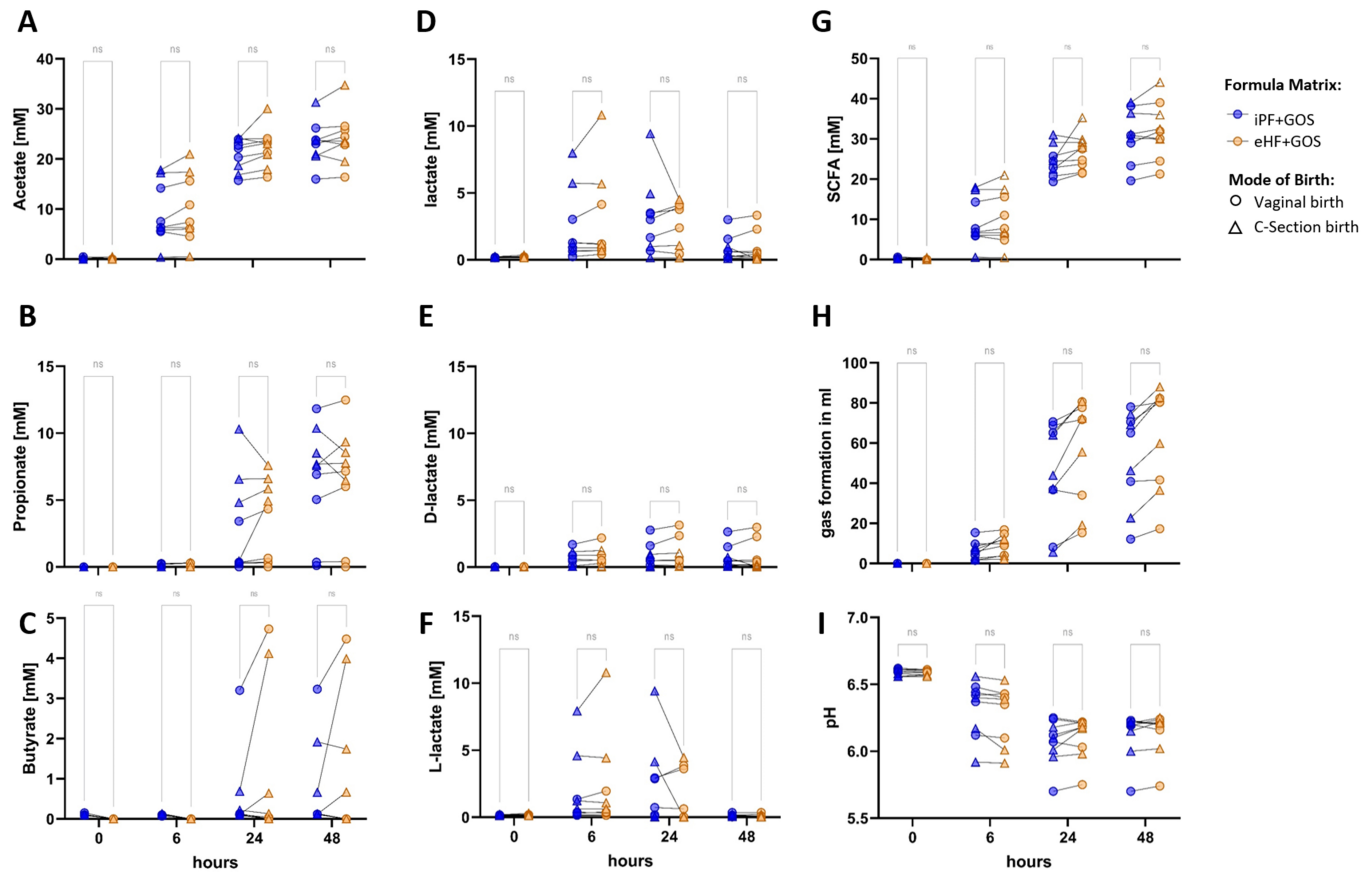
Effect of formula protein matrix on the metabolic activity of infant fecal donor microbiota. (A) Acetate; (B) propionate; (C) butyrate; (D) total lactate; (E) D-lactate; (F) L-lactate; (G) total SCFA production; (H) pH; and (I) volume of gas production during simulated colonic fermentation with donor infant microbiota and either iPF (blue) or formula with eHF (orange). Both formulae contained prebiotic GOS. Samples from the same donor are linked with a line. Donor samples of infants born by caesarian section are shown as triangles, and donor samples of infants born via VD mode are shown as circles. n.s.: Not significant; iPF: intact protein formula; GOS: galactooligosaccharide; eHF: extensively hydrolyzed formula; SCFA: short-chain fatty acid.

To investigate the effects of an intact and hydrolyzed protein formula matrix on the donor ecosystem, the SHIME^®^ model was inoculated with fecal suspension of nine exclusively breastfed infants. Subsequently, iPF + GOS or eHF + GOS formula was added to the system. Acetate levels were rising within the first 6 h of colonic fermentation, and a trend toward higher acetate levels in eHF + GOS was observed for all inocula compared to iPF + GOS (*P* > 0.05; Figure 2A).

Propionate and butyrate levels increased at later time points during colonic incubations. High inter- and intra-individual variations were observed and these were not influenced by the type of formula or birth mode [Figure 2B and C]. Total lactate levels were already increasing at 6 h and decreased again over time, independent of the formula matrix [Figure 2D]. D-lactate was also formed by the infant donor microbiota [Figure 2E]. In contrast, L-Lactate was almost depleted by 48 h of colonic fermentation, showing cross-feeding mechanisms in the complex bacterial ecosystem [Figure 2F]. In summary, the formation of total SCFA increased over time, with acetate as the quantitatively most abundant SCFA produced by the microbiota of exclusively breastfed infants [Figure 2G]. All samples showed only a non-significant trend toward slightly increased SCFA levels (*P* > 0.05) in eHF + GOS, independent of birth mode. Increased SCFA formation is also mirrored in a drop in pH with infant microbiome inoculum [Figure 2H]. Formation of gas by the un-supplemented (i.e., without probiotic supplementation) microbiota showed a wide range between the individuals and increased with time and was independent of the formula matrix and mode of birth [Figure 2I].

### Effect of probiotic supplementation on the metabolic activity of an *ex-vivo* infant intestinal microbial ecosystem

**Figure 3:**
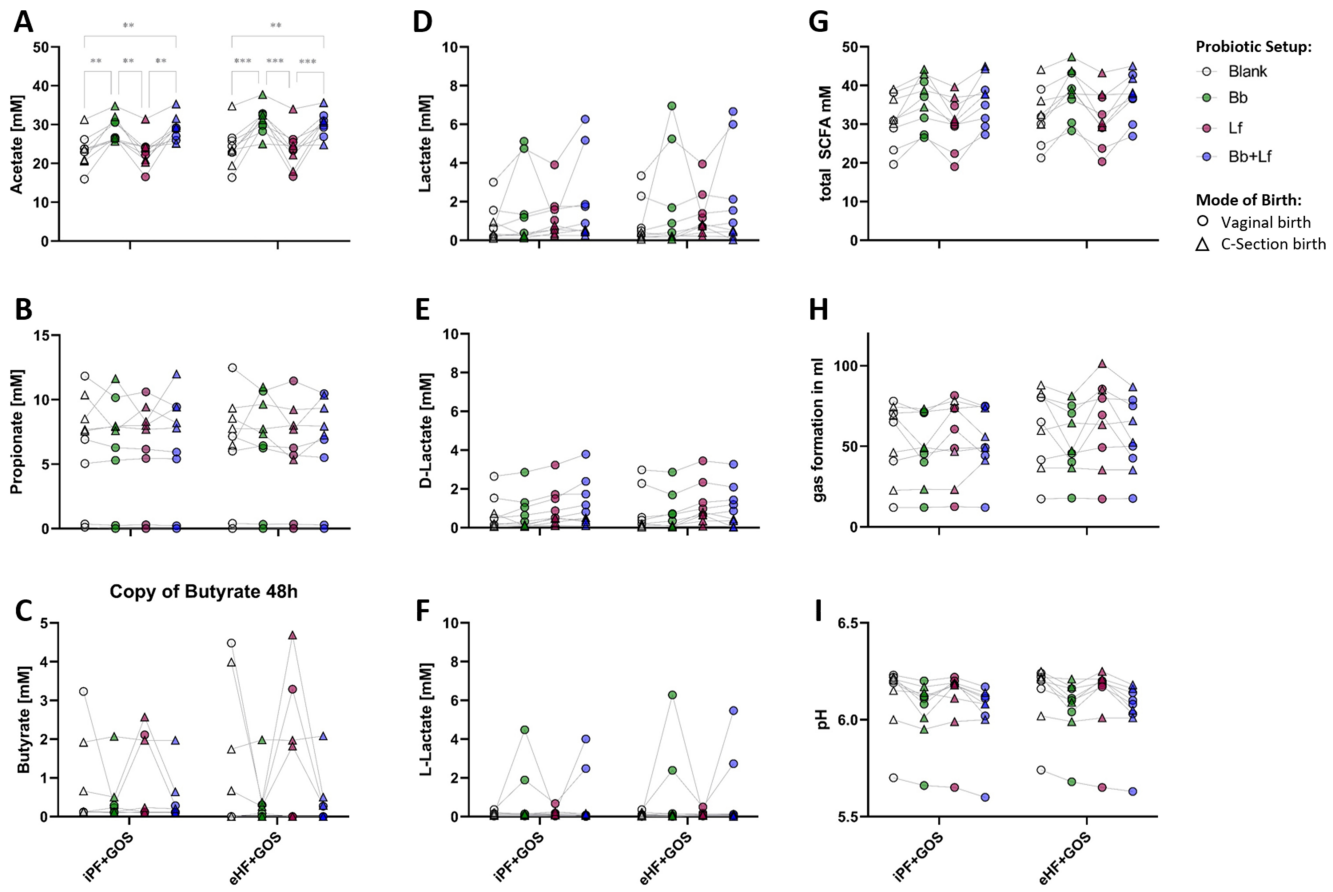
Probiotic strain-specific effects on the metabolic activity of infant donor microbiota in the SHIME^®^ model. (A) Acetate; (B) Propionate; (C) Butyrate; (D) total lactate; (E) D-lactate; (F) L-lactate; (G) total SCFA production, (H) pH and (I) volume of gas production during simulated colonic fermentation with donor infant microbiota and either intact protein formula (iPF + GOS) or formula with extensively hydrolyzed protein matrix (eHF + GOS). Selection of significant differences are marked with a ^*^P < 0.05, ^**^P < 0.01, or ^***^P < 0.001. Probiotic strains were added in a concentration of 1.5 × 10^7^ CFU/mL each. SCFA: Short-chain fatty acid; Blank: no probiotic strain added to the donor microbiota; Bb: *B. breve* DSM 32583; Lf: *L. fermentum* CECT 5716; Bb + Lf: both probiotics added; iPF: intact protein formula; GOS: galactooligosaccharide; eHF: extensively hydrolyzed formula; SHIME: Simulator of the Human Intestinal Microbial Ecosystem.

Changes in the bacterial ecosystem are associated with changes in the overall metabolic activity. Therefore, changes in intestinal metabolites upon probiotic supplementation of formulae with either intact or hydrolyzed protein were assessed [Figure 3]. The simulated colonic incubation was done with either single probiotic supplementation [Bb (green) or Lf (grey)] or double probiotic supplementation (Bb + Lf; blue). Values of un-supplemented conditions (samples shown in Figure 2A-I) are shown again for comparison (Blank; white).

The formation of individual SCFA seemed to be strain-specific. Supplementation with Bb or the combination of Bb + Lf yielded significantly higher levels of acetate, compared to non-supplemented (Blank) or Lf-supplemented conditions [Figure 3A]. Levels were independent of birth mode, as seen by similar distribution of children born by VD (circles) and CS (triangles). In contrast, patterns for propionate, butyrate, and lactate (neither D- nor L-lactate) showed high individual variation and did not differ between the added probiotics [Figure 3B-F]. Overall, Bb and Bb + Lf supplementation showed a tendency to increase the total amount of SCFA, independent of the formula matrix, compared to respective controls (*P* > 0.05) [Figure 3G]. Production of different acids seems to reduce pH levels, though changes were not significant after the observed time window (*P* > 0.05). Interestingly, while probiotic addition seemed to increase the gas production *in vitro*, in the presence of a complex microbiota, levels of gas formation were comparable between supplemented and control conditions and probiotic addition did not increase the amount of gas production (*P* > 0.05) [Figure 3H and I].

### Combined effects of formula matrix and probiotic supplementation on the donor infant intestinal microbiota

Supplementation with probiotics showed effects on the overall metabolic activity in the SHIME^®^ model. Therefore, the effect on the microbial composition was also assessed. At the end of colonic incubation, samples were taken from all reactors (nine infant donors), and for five infants, the microbiome was analyzed by shotgun metagenome sequencing. The specific infant gut database (Version1 including the WGSs of Bb and Lf) and complementary bioinformatics pipeline were used to produce taxonomic profiles of the metagenome data. The average percentage classified for all samples was 98.6% ± 0.97% at the genus level.

**Figure 4:**
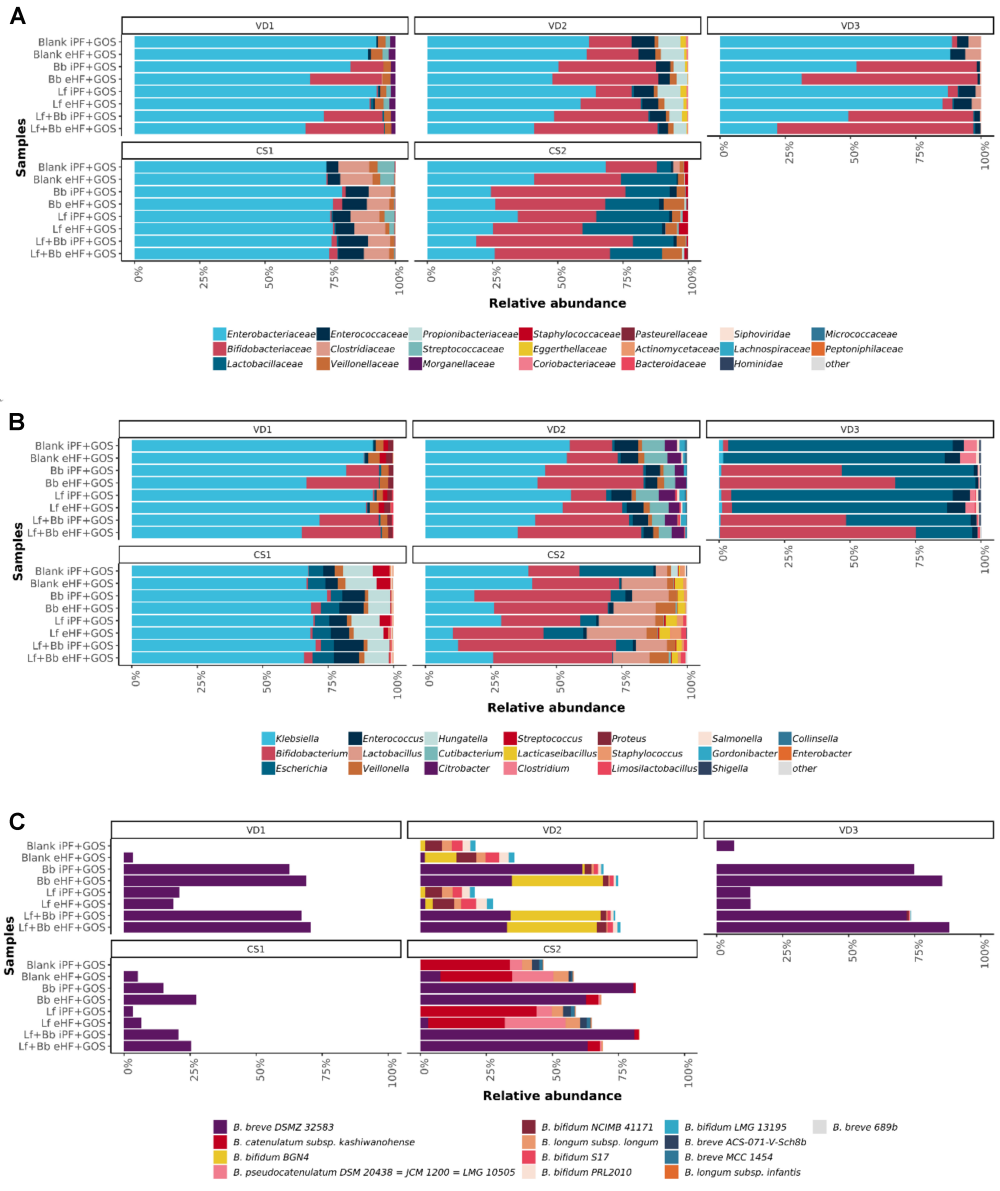
Infant microbiome composition in the SHIME^®^ gut model. (A) Family level taxonomic profile; (B) Genus level taxonomic profile; (C) Relative abundance of all detected *Bifidobacterium* strains from samples after 48 h in the *ex vivo* fermentors for 8 different media compositions for each infant fecal inoculum. Blank: No probiotics; iPF: intact protein formula; GOS: galactooligosaccharides; eHF: extensively hydrolyzed formula; Bb: *B. breve* DSM 32583; Lf: *L. fermentum* CECT 5716; Lf + Bb: both probiotics added; VD: fecal inoculum derived from vaginally delivered infant; CS: fecal inoculum derived from cesarean-delivered infant; SHIME: Simulator of the Human Intestinal Microbial Ecosystem.

Probiotic strains in IF have to survive the gastrointestinal passage and ideally thrive on the co-administered prebiotic compounds (e.g, GOS). Both strains survived the small intestinal passage at a comparable level (data not shown). Upon colonic fermentation for 48 h, small differences in the overall composition of the donor microbiota composition were observed for infants born via VD or CS [Figure 4A-C]. Family-, Genus-, Species-, and Strain-level relative read abundance profiles were generated using shotgun metagenomics sequencing. Though all infants were of approximately the same age and all were exclusively breastfed, high inter-individual variability was observed in microbial diversity, as expected for this age group [Figure 4], such as the difference in abundances of *Bifidobacteriaceae* and *Clostridiaceae* [Figure 4A]. Small effects of the formula matrix can be seen in some infants, but not in others.

The presence of Bb significantly reduced the mean relative abundance of the genus *Salmonella* within the family *Enterobacteriaceae* (*P* = 0.0045, *P* = 0.0057), the genus *Streptococcus* within the family *Streptococcaceae* (*P* = 0.0091, *P* = 0.0098) and the family *Lachnospiraceae* (*P* = 0.0044). The reduction of these genera and families was associated with a reduction in virulence genes in the metagenome^[40]^.

### Probiotic abundance in the *in vitro* colon model after 48 h of fermentation

**Figure 5:**
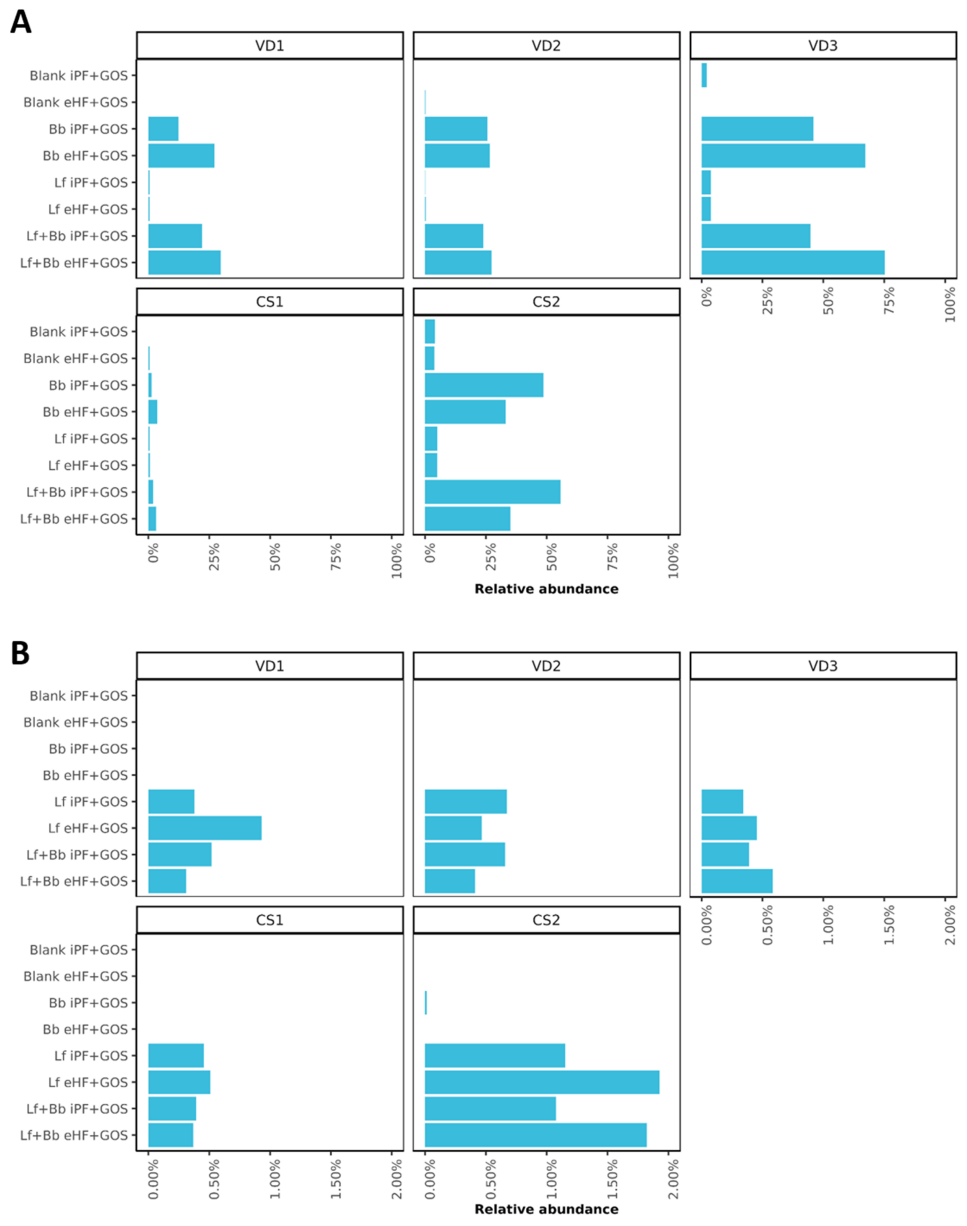
Probiotic strain abundances in the SHIME^®^ model. (A) High inter-individual differences in relative abundance of Bb; (B) Relative abundance of Lf. From samples after 48 h in the *ex vivo* SHIME^®^ model for each infant fecal inoculum. Blank: No probiotics; iPF: intact protein formula; GOS: galactooligosaccharides; eHF: extensively hydrolyzed formula; Bb: *B. breve* DSMZ 32583; Lf: *L. fermentum* CECT 5716; Lf + Bb: both probiotics added; VD: fecal inoculum derived from vaginally delivered infant; CS: fecal inoculum derived from cesarean-delivered infant; SHIME: Simulator of the Human Intestinal Microbial Ecosystem.

The analysis pipeline with a specific infant database, as created here, enables taxonomic classification at the strain level in infant microbiota samples. This approach was applied to track the probiotic strains in the microbiota samples from the *ex vivo* model. The abundances of the probiotic strains supplemented in this study are shown in Figures 5A and B. The observed abundance of *Bifidobacteriaceae* (relative abundance average 33.80%) in the infant donor microbiota was much higher than *Lactobacillaceae* (relative abundance average 4.47%) despite the same amount of supplementation. Each of the probiotics was detected in the samples where it was supplemented, as expected.

In addition to the supplemented Bb strain, the infant microbiota also contained commensal bifidobacterial species [Figure 4C]. In donor VD2 and donor CS2, a non-significant shift after the addition of the probiotic strain was observed (light blue). The addition of Bb replaces commensal strains (e.g., *B. catenulatum* in CS2) while promoting the growth of others (e.g., *B. bifidum* BGN4 in VD2). Interestingly, in all infants except for CS2, Bb abundance was higher in combination with the eHF-GOS formula, despite its lower growth rates in the *in vitro* setup. This suggests possible formula matrix-dependent effects as well as cross-feeding mechanisms with other members of the infant gut microbiota. This is observed when Bb is added both alone and in combination with Lf.

### Addition of Bb indicates effects on the infant microbial community *ex vivo*

**Figure 6:**
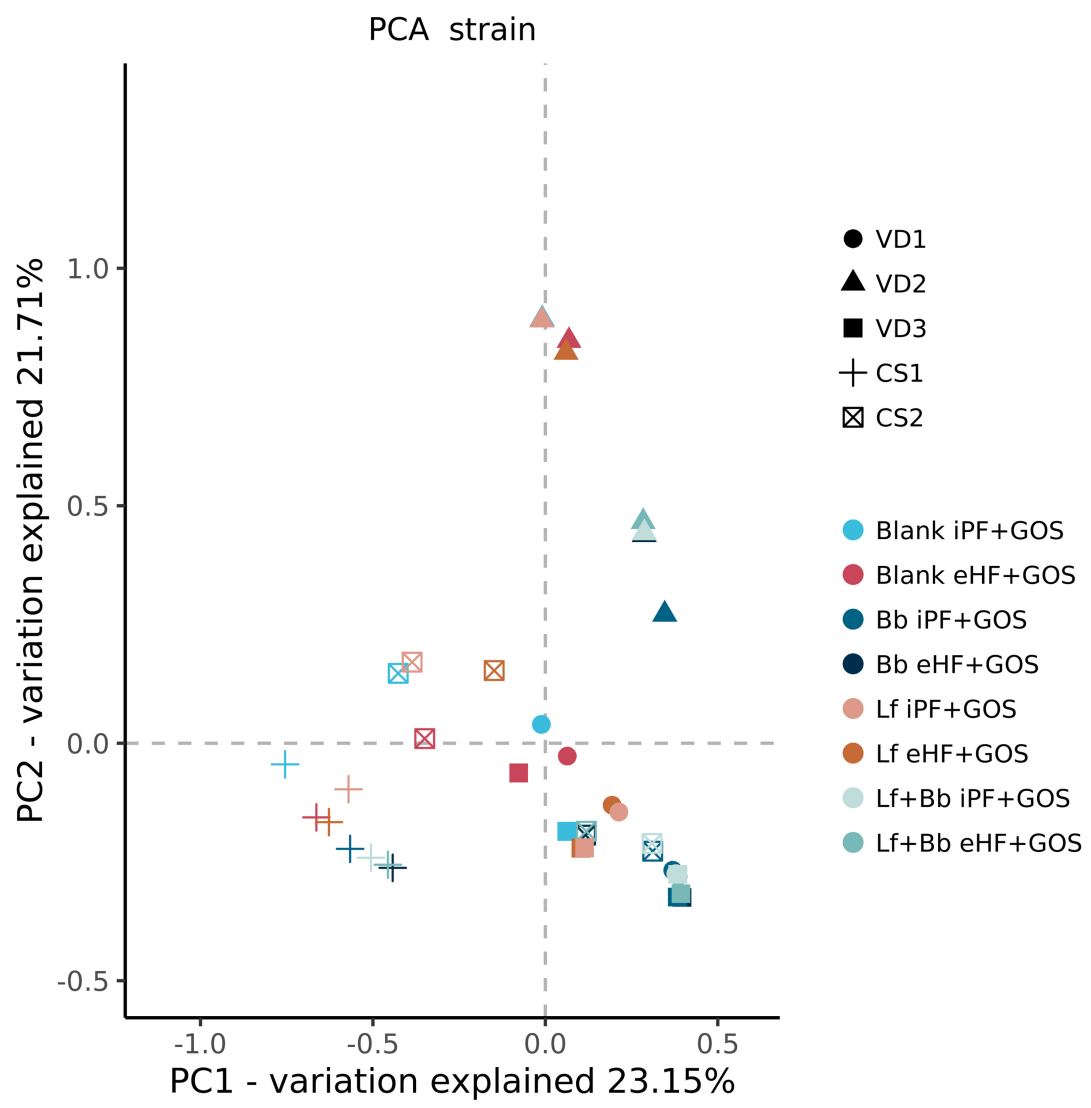
PCA analysis at the strain level from samples after 48 hours in the *ex vivo* model of the infant bacterial ecosystem. PCA: Principle Coordinate Analysis; VD: fecal inoculum derived from vaginally delivered infant; CS: fecal inoculum derived from cesarean-delivered infant; Blank: no probiotics; iPF: intact protein formula; GOS: galactooligosaccharides; eHF: extensively hydrolyzed formula; Bb: *B. breve* DSMZ 32583; Lf: *L. fermentum* CECT 5716; Lf + Bb: both probiotics added.

Fermentation for 48 h showed a trend toward an increase in bifidobacteria in all infants, independent of the formula matrix [Figure 4C and Figure 5A], especially when supplemented with probiotic Bb; however, changes were not significant (*P* > 0.05) [Figure 4A]. Interestingly, strain-level detection of Bb did show a significant increase due to probiotic supplementation *vs.* no Bb supplementation. Bb-strain abundance (27.31 median relative abundance) showed a significant (*P*-value < 0.005) shift from the samples without Bb (0.46 median relative abundance), which is not observed with Lf supplementation. Interestingly, both probiotics were added in the same amount. Principle Coordinate Analysis (PCA) was performed [Figure 6] to visualize the impact of both probiotics on the overall microbiota. PERMANOVA analysis of samples containing Bb showed a significant (*P*-value = 0.005) shift from the samples without Bb, which was not observed with Lf supplementation alone, thereby consolidating the previous observation.

## DISCUSSION

Our results show that *in vitro*, hydrolyzed infant formula (eHF) appears to have a strong influence on the metabolic activity of probiotic strains. Interestingly, in the presence of a complex microbial ecosystem in an *ex vivo* infant gut model, no effect on the metabolic activity of the microbiota was observed. However, strain-dependent effects (e.g., acetate production) were still found *in vitro* and *ex vivo*, and therefore even in complex ecosystems, but independent of the formula matrix.

Nevertheless, high inter-individual differences in the donor microbiota composition and probiotic abundance were observed in the complex ecosystem and could be demonstrated at the strain level.

Breastfeeding is the best possible nutrition for infants. Isolating probiotic strains from HM is a potential method to transfer this HM-mediated protection to children who cannot be breastfed. Bb was isolated from HM and recently assessed for probiotic health effects and safety^[41]^. Lf, which was isolated from HM many years ago, has demonstrated health-promoting mechanisms in preclinical and human studies^[42]^. Recently, it was shown in a large infant cohort that the combination of Lf and GOS could modify the overall infant microbiota composition closer to the breastfed reference group at the age of four months^[43]^. While only being present in low abundance as shown here, the administration of Lf was shown to exert positive effects on respiratory pathologies^[44]^ despite low abundance in the stool microbiota^[43]^ in this cohort. Positive effects on health outcomes despite the low abundance of the probiotics in the microbiota have been observed for this strain by others before^[45]^.

The addition of probiotics to food and the associated microbial activity and generation of SCFA can be associated with gas formation, especially in combination with prebiotic compounds^[46,47]^. Interestingly, here, the combination of two probiotic strains abolished the gas formation found for a single probiotic *in vitro*, suggesting cross-feeding mechanisms. These cross-feeding mechanisms might also be seen through the enantioselective degradation of L-Lactate throughout the observation period^[48,49]^. While these effects were significant *in vitro*, no effects were visible anymore in the presence of a complex host microbiota, indicating the involvement of other members of the donor microbiota in these cross-feeding mechanisms.

Deciphering the presence of a probiotic strain in a complex infant gut microbiome at strain-level resolution is a challenge. In a previous study on the development of the infant microbiota^[50]^, analysis of strain-level resolution was used to demonstrate the transfer of microbes between mother and infant. Strain-level taxonomic profiling is also important for the medical and pharmaceutical field, since research has shown that strains belonging to the same species can have very different phenotypes and functional properties^[51-53]^. Finally, the food industry has a high interest in analyzing ecosystem data on a strain-level resolution to demonstrate the possible success of a probiotic product and its temporal persistence in the gut. The use of a validated infant-specific database for taxonomic profiling of the samples made it possible to identify members of the microbiota at the strain level. This knowledge is important for the development of health-promoting probiotics and the evaluation of their performance in different matrices.

Infant formula based on partially or extensively hydrolyzed protein has been used for many years for infants at risk of allergy development who cannot be exclusively breastfed^[12,54-56]^. However, there is still uncertainty regarding the mechanism behind this assumed protection^[56]^. Recently, the presence of already pre-hydrolyzed protein in the IF raised the question of an effect on the overall microbial ecosystem and subsequent effects on the immune maturation of the infants^[57]^. Especially for infants in their first year of life, the microbiota is still developing and has not yet reached an equilibrium. Therefore, a clear definition of a healthy microbiota for this age group is difficult. However, bacterial species from the family of *Lactobacillaceae* and *Bifidobacteriaceae* are assumed markers of a healthy microbiota and are often added to infant formula or food supplements^[58-60]^. While the strain-specific effects of probiotics are well known, knowledge about the effect of the surrounding - i.e., co-administered - matrix is scarce. IF with hydrolyzed protein is tested for its suitability to support infant’s growth, as well as putative allergy preventative effects. Recently, the allergenic activity of Lf in the respective formulae was tested *in vitro* in a basophil activation experiment, revealing attenuated basophil degranulation in combination with eHF. As this attenuation was absent in the combination with iPF, the protein matrix might have a significant effect on the metabolic activity and survival of probiotic strains^[17]^.

The present study showed that the eHF induced only minor effects on the overall composition of the bacterial ecosystem in the *ex vivo* model. One reason might be the presence of prebiotic GOS in both matrices, which might be overriding any minor effects based on the hydrolyzed protein per se. A recent study by Heppner *et al.* also showed the more pronounced effect of GOS supplementation compared to probiotic supplementation^[61]^.

There are several limitations to be considered when using *ex vivo* models with complex microbial ecosystems and food matrices to characterize the effects of food composition on the gut ecosystem and host health. The biggest limitation can be seen in the one-time dosage of the probiotics as well as infant formulae to the *ex vivo* model. While gut models surpass the issue of compliance and confounders of human intervention trials, the long-term effects of repeated and continuous application of pro- and prebiotics are still a challenge. Therefore, assessing higher numbers of infant microbiota donors and assessing them at several different time points and after repeated application of the infant formula might have given a more realistic model and should be tested in the future. The infant gut microbiota is known to be very variable, so a larger number of donors would be needed, as each infant has an individual response toward the different probiotics and protein matrices used in this study. Particularly at this early age, the mode of birth can also have a significant effect on the composition of the microbiome and, therefore, on probiotic colonization and activity. Therefore, a larger number of infants born either vaginally or by CS would be needed to elucidate the mode-specific effects in order to draw more reliable conclusions. In addition, a greater range in age of the infants providing the donor microbiota and more variety in their feeding history (i.e., exclusively breastfed, exclusively formula fed or mixed feeding of HM and IF) would help to elucidate the ecosystem-specific dynamics in more detail. In the present study, only exclusively breastfed infant donor samples were used. Therefore, the donor microbiota was adapted to the presence of different HM oligosaccharides from the previous feeding history. Therefore, the adaptation of a complex bacterial ecosystem from infants fed exclusively with IF without prebiotic compounds would be of interest for future studies. In addition, both infant formulae contained the prebiotic compound GOS to support health-associated bacteria in the infant donor microbiota, as well as the added probiotic compound. A complex IF with the addition of probiotics - but without the presence of prebiotics - to the respective formulas would have been of interest to see possibly greater effects of the respective protein matrix. In the present study, the single or combined supplementation with infant-age-associated probiotics was tested. When the probiotic strains were used in combination, a 1:1 ratio was used. Future studies should also test the effects of different ratios or total numbers of probiotics on metabolic activity to further elucidate putative synergistic effects.

While sequencing-based methods have improved significantly in their ability to detect different members of the microbial ecosystem, their main limitation is still their blindness to the microbial load in the ecosystem and their inability to discriminate between viable or metabolically inactive and dead bacteria. Therefore, methods are needed that enable the enumeration of microbial cells with the ability to reliably discriminate between viable and dead members of the microbiota. These future approaches, such as the combination of amplicon sequencing and fluorescence-activated cell sorting, will improve the elucidation of the interplay between the microbial ecosystem and host health.

Microbial colonization of the neonatal gut is an essential event, as the establishment of a healthy gut microbiota is pivotal in priming the infant’s immune system and modifying the risk for infections or allergies in later life. When exclusive breastfeeding is not possible, IF with all its different compounds will have a major impact on the infant’s microbiome maturation. Therefore, a good understanding of the effects of different IF compounds on the microbiome and supplemented probiotics is important.

The infant gut microbiome database and the bioinformatics pipeline reliably identified the microbiome of the samples at the strain level. This enables the assessment of the effects of the different formula matrices and probiotic supplements on the probiotic abundance and overall bacterial composition. With this experimental setup, i.e., a combination of *ex vivo* digestive models, shotgun metagenomics analysis, and a specific bioinformatics pipeline, the presence of the supplemented Bb and Lf strains was detected.

The present work highlights the crucial finding that the infant microbiota is highly variable and may be influenced in various ways by different infant formulae and their components, in the event that exclusive breastfeeding is not possible. Further studies are required to demonstrate the effects of different food compounds on supplemented probiotics and the infant microbiome at this early and pivotal time point for a healthy microbiome and immune maturation.

## DECLARATIONS

### Copyright

© The Author(s) 2024.
